# Discriminative pattern of reduced cerebral blood flow in Parkinson’s disease and Parkinsonism-Plus syndrome: an ASL-MRI study

**DOI:** 10.1186/s12880-020-00479-y

**Published:** 2020-07-13

**Authors:** Lina Cheng, Xiaoyan Wu, Ruomi Guo, Yuzhou Wang, Wensheng Wang, Peng He, Hanbo Lin, Jun Shen

**Affiliations:** 1 Department of Radiology, Sun Yat-Sen Memorial Hospital, Sun Yat-Sen University, Guangzhou, 510120 China; 2 Department of Radiology, Guangdong 999 Brain Hospital, Guangzhou, 510510 China; 3 Department of Radiology, The Third Affiliated Hospital, Sun Yat-Sen University, Guangzhou, 510630 China; 4 Department of Neurology, Guangdong 999 Brain Hospital, Guangzhou, 510510 China

**Keywords:** Parkinson’s disease, Parkinsonism-plus syndrome, Magnetic resonance imaging, Arterial spin labeling

## Abstract

**Background:**

Accurate identification of Parkinson’s disease (PD) and Parkinsonism-Plus syndrome (PPS), especially in the early stage of the disease, is very important. The purpose of this study was to investigate the discriminative spatial pattern of cerebral blood flow (CBF) between patients with PD and PPS.

**Methods:**

Arterial spin labeling (ASL) perfusion-weighted imaging was performed in 20 patients with PD (mean age 56.35 ± 7.56 years), 16 patients with PPS (mean age 59.62 ± 6.89 years), and 17 healthy controls (HCs, mean age 54.17 ± 6.58 years). Voxel-wise comparison of the CBF was performed among PD, PPS, and HC groups. The receiver operating characteristic (ROC) curve was used to evaluate the performance of CBF in discriminating between PD and PPS. The relationship between CBF and non-motor neuropsychological scores was assessed by correlation analysis.

**Results:**

PD group showed a significantly decreased CBF in the right cerebelum_crus2, the left middle frontal gyrus (MFG), the triangle inferior frontal gyrus (IFG_Tri), the left frontal medial orbital gyrus (FG_Med_Orb) and the left caudate nucleus (CN) compared with the HC group (*P* < 0.05). Besides the above regions, the left supplementary motor area (SMA), the right thalamus had decreased CBF in the PPS group compared with the HC group (*P* < 0.05). PPS group had lower CBF value in the left MFG, the left IFG_Tri, the left CN, the left SMA, and the right thalamus compared with the PD group (*P* < 0.05). CBFs in left IFG_Tri, the left CN, the left SMA, and the right thalamus had moderate to high capacity in discriminating between PD and PPS patients (AUC 0.719–0.831). The CBF was positively correlated with the Mini-Mental State Examination (MMSE) and Montreal Cognitive Assessment (MoCA) scores in PD patients, while positively correlated with the MMSE, Hamilton Anxiety Scale (HAMA), Hamilton Depression Scale (HAMD) scores in PPS patients (*P* < 0.05).

**Conclusion:**

PD and PPS patients have certain discriminative patterns of reduced CBFs, which can be used as a surrogate marker for differential diagnosis.

## Background

Parkinson’s disease (PD) and Parkinsonism-Plus syndrome (PPS) are clinically common chronic progressive neurodegenerative diseases in middle-aged and senior population. The known pathological basis of PD is over 50% reduction of dopaminergic neurons in the substantia nigra pars compacta of the midbrain, resulting in a decrease of dopamine production and a relative increase of antagonistic neurotransmitter acetylcholine, which causes hyperfunction. PPS includes a variety of diseases, such as multiple system atrophy (MSA), progressive supranuclear palsy (PSP), and corticobasal degeneration (CBD). The common feature is the implication of the extrapyramidal system [1–3]. Albeit similar symptoms, clinical treatment for PD and PPS is obviously different [4–6]. Thus, accurate identification of these two types of diseases, especially in the early stage of the disease, is very important [7, 8].

Previously, some brain structural changes on magnetic resonance imaging (MRI) have been shown to be able to differentiate between PD and PPS, such as a cross sign, fissure sign, hummingbird sign, swallow tail sign [9–11]. Unfortunately, these signs mainly occur in the later stage of diseases. Positron emission tomography (PET) and single-photon emission computed tomography (SPECT) can be used for early identification by observing metabolic changes in the brain region. However, it is not widely available and suffers from shortcomings of high cost and radiation hazards [12, 13]. Besides structural MRI, functional MRI has additional value for differential diagnosis [14–18]. Among these functional imaging, arterial spin labeling (ASL)-based perfusion-weighted imaging is a novel, non-invasive method that does not require the injection of contrast agent. Currently, three-dimensional pseudo-continuous arterial spin labeling (3D-pCASL) has been used to measure cerebral perfusion in neurodegenerative diseases quantitatively [19] and demonstrated abnormal cerebral perfusion in PD and MSA [20, 21].

However, whether the spatial pattern of cerebral blood flow (CBF) as measured by ASL imaging is different between patients with PD and PPS remains unclear. In this study, 3D-pCASL was performed in PD and PPS patients to investigate the discriminative spatial pattern of CBF abnormalities in patients with PD and PPS.

## Methods

### Subjects

From Jan 2017 to Jan 2019, consecutive patients who were clinically diagnosed with PD and PPS were enrolled. The diagnosis of PD was confirmed according to the Clinical Diagnostic Criteria for Parkinson’s Disease in China (2016) [22], and current consensus clinical criteria for PPS [2]. The exclusion criteria were as follows: cerebral hemorrhage, infarction, brain tumors, trauma, severe white matter hyperintensity, contraindication to MRI, and image quality of structural or ASL imaging ineligible for data analysis. Finally, 20 PD patients including 13 males and 7 females, with a mean age of 56.35 ± 7.56 years (range, 43 to 66 years), and 16 PPS patients including 7 males and 9 females, with a mean age of 59.62 ± 6.89 years (range, 41 to 66 years) were enrolled. 17 healthy controls (HCs) were collected, including 6 males and 11 females, with a mean age of 54.17 ± 6.58 years (range, 44 to 65 years). Non-motor neuropsychological scores including Hamilton Anxiety Scale (HAMA), Hamilton Depression Scale (HAMD), Mini-Mental State Examination (MMSE), and Montreal Cognitive Assessment (MoCA) were obtained in PD and PPS patients. All neuropsychological evaluations and MRI were performed under medication in “on” state, similar to a previous study [23].

### MRI acquisition

All participants underwent an MRI examination on a 3.0 T scanner (Signa HDxt, GE Medical Systems) with an 8-channel, phased-array head coil. The sequences included axial three-dimensional brain volume T1-weighted imaging (repetition time [TR] = 8.8 ms, echo time [TE] = 3.5 ms, inversion time [TI] = 450 ms, flip angel [FA] = 13°, Matrix = 320 × 320, field of view [FOV] = 240 × 240 mm, number of excitations = 1, slice thickness = 1.2 mm, slice gap = 0 mm) and 3D pCASL. 3D-pCASL images were acquired by using a spiral fast spin echo sequence (TR/TE, 4599 ms/9.8 ms; matrix = 512 × 512; FOV = 240 × 240 mm; number of excitations, 3; slice thickness, 4 mm; and post-labeling delay time, 1525 ms).

### Image analysis

All ASL data was transferred to the AW4.6 workstation (GE Healthcare) to generate CBF maps by using GE functool 4.6 software. The calculation of the CBF was based on the following equation [24], $\mathrm{CBF}\ \left[\mathrm{mL}/100\mathrm{g}/\min \right]=\frac{6000\times \lambda \times \left({SI}_{control}-{SI}_{label}\right)\times {e}^{\frac{PLD}{T_{1, blood}}}}{2\times \alpha \times {T}_{1, blood}\times {SI}_{PD}\times \left(1-{e}^{\frac{-\tau }{T_{1, blood.}}}\right)}$

where λ (blood-brain partition coefficient) = 0.9 mL/g, T_1, blood_ at 3.0 Tesla (the longitudinal relaxation time of blood) = 1650 ms, α (labeling efficiency) for pCASL = 0.85, τ (labeling duration) = 1500 ms, SI_control_ and SI_label_ are the time-averaged signal intensities in the control and labeled images, SI_PD_ is the signal intensity of a proton density-weighted image. CBF map was preprocessed by using statistical parametric mapping (SPM, http://www.fil.ion.ucl.ac.uk/spm/software/spm12) based on Matlab2013a platform. Voxel-based analysis (VBA) was applied in this study and the specific processing was similar to those in the literature [25, 26]. At first, the CBF map and 3D T1WI map were manually reoriented to Montreal Neurological Institute (MNI) space and centered on anterior commissure for the following segmentation and spatial normalization. The T1WI map was co-registered to CBF image using a ridged-body model [27]. Voxel-to-voxel affine transformation matrix was generated and written into the T1WI map. Then, the T1WI map was segmented into gray matter, white matter, cerebrospinal fluid by using a unified segmentation algorithm [28]. CBF map was further spatially normalized to standard MNI space (resampling voxel size = 2 mm × 2 mm × 2 mm) with the segmentation information. Finally, quality control was conducted to exclude patients with bad normalization. Spatial smoothing was performed for the normalized CBF map to increase the signal-to-noise ratio (SNR) with a 4 mm full-width at half-maximum (FWHM) isotropic Gaussian kernel.

### Statistical analysis

One-way analysis of variance (ANOVA) or *χ*^*2*^-test was used to evaluate the difference in age and gender among the three groups. Two-sample *t*-test was applied to compare the differences in disease duration, HAMA, HAMD, MMSE, and MoCA scores between PD and PPS groups. ANOVA was used to identify significant between-group differences in the CBF map among the three groups. Correction for multiple comparisons was performed using Gaussian Random Field (GRF) correction with a voxel-level threshold at *p* < 0.01, and cluster-level *p* < 0.05. The Anatomical Automatic Labeling (AAL) brain template was used to report brain regions. The CBF of brain regions with statistically significant difference were extracted for each subject. A post-hoc ANOVA with the least significant difference test (LSD) was used to identify the significance of pair-wise group (PD vs. PPS, PD vs. HC, and PPS vs. HC) differences in CBF value (*p* < 0.05). Receiver operating characteristic (ROC) analysis was used to evaluate the performance of CBF from significant brain regions in differentiation between the PD and PPS groups. Pearson’s correlation was performed to evaluate the relationship between CBF values and clinical scores in PD and PPS groups. A *p* value of less than 0.05 was considered as statistically significant. Statistical analyses were performed by using the SPSS (version 26.0; SPSS, Chicago, III., USA).

## Results

### Characteristics of study population

The clinical and demographic information of the PD and PPS patients are shown in Table 1, numerical variables were presented as mean ± standard deviation. All PPS patients initially developed Parkinson’s symptoms, including 5 cases of MSA, 2 cases of PSP, 1 case of CBD, and 8 cases of unclassified PPS. There were no statistical differences in gender and age among the PD, PPS, and HC groups (*p* > 0.05). The PD and PPS groups had a significant difference in disease duration (*p* = 0.02). Compared with PD patients, PPS patients had significantly lower MMSE scores (*p* = 0.001). There were no differences in the HAMA, HAMD, and MoCA scores between PD and PPS patients (*p* > 0.05).

**Table 1:** Demographic and clinical characteristics of the participants

Characteristics	PD (*n* = 20)	PPS (*n* = 16)	Control (*n* = 17)	*p*-Value
Age, years	56.35 ± 7.56	59.62 ± 6.89	54.17 ± 6.58	0.094^a^
Gender, male/female	13/7	7/9	6/11	0.174^b^
Disease duration years	6.40 ± 4.04	3.50 ± 2.81	NA	0.020^c^
HAMA scale	16.50 ± 5.92	13.36 ± 7.85	NA	0.145^c^
HAMD scale	18.45 ± 6.91	16.09 ± 9.30	NA	0.546^c^
MMSE scale	26.05 ± 3.26	19.25 ± 8.87	NA	0.001^c^
MoCA scale	20.55 ± 4.60	13.41 ± 7.69	NA	0.231^c^

**Abbreviations:**^a^ One-way ANOVA; ^b^ Chi-square (*χ*^*2*^) test; ^c^ Two-sample *t*-test; PD, Parkinson’s disease; PPS, Parkinsonism-Plus syndrome; HAMA, Hamilton Anxiety Scale; HAMD, Hamilton Depression Scale; MMSE, Mini-Mental State Examination; MoCA, Montreal Cognitive Assessment

### CBF in PD, PPS and HC groups

**Fig. 1:**
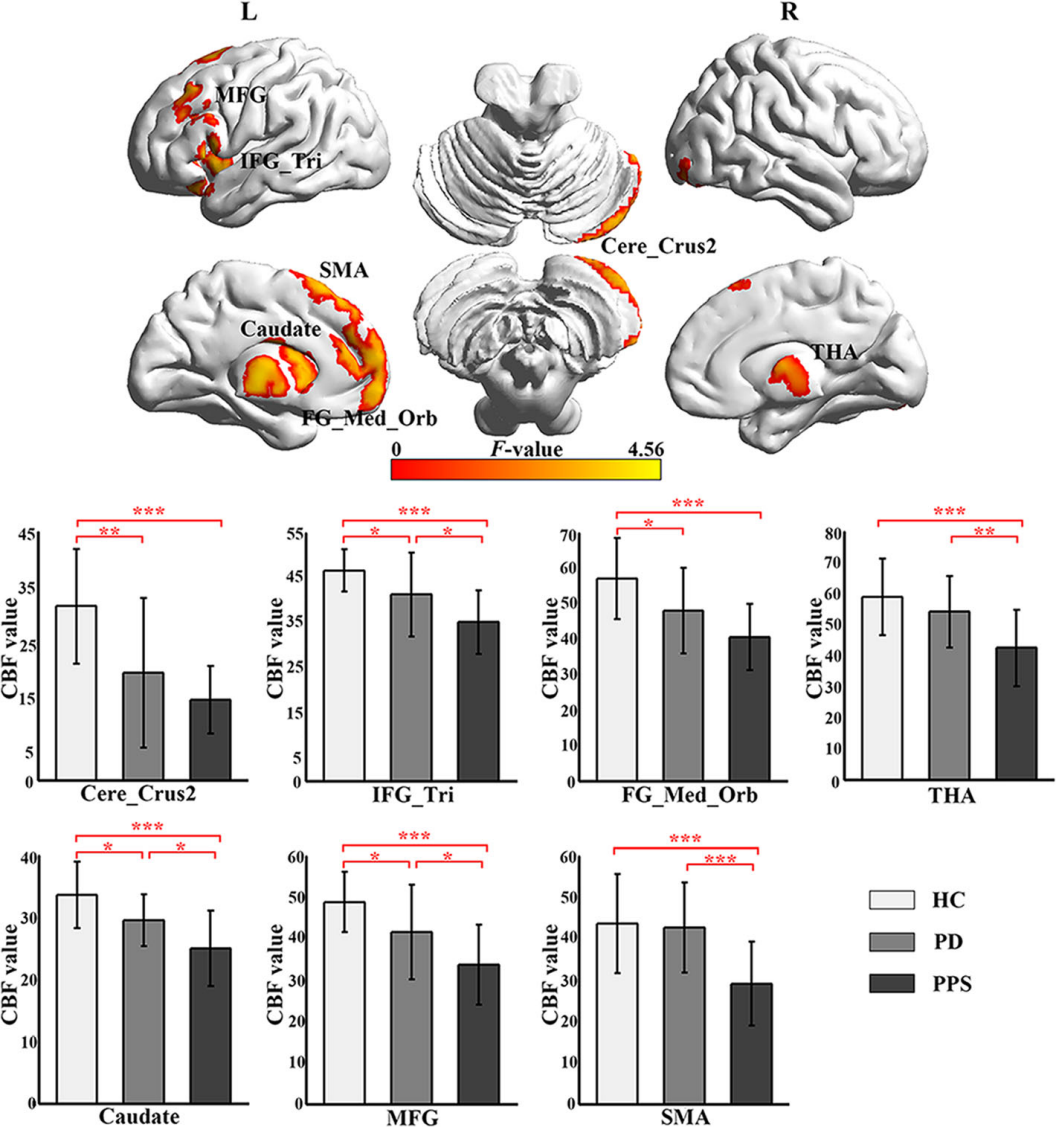
The significant differences of CBF between the PD, PPS, and HC groups. (a) Brain regions of CBF differences between the PD, PPS, and HC groups. (b) The bar plot shows the pair-wise contrasts in CBF value between the three groups. The bar height corresponds to the mean value of the CBF and the error bar to the standard deviation for each group. Abbreviations: SMA, supplementary motor area; THA, thalamus; FG_Med_Orb, frontal medial orbital gyrus; IFG_Tri, triangle inferior frontal gyrus; MFG, middle frontal gyrus; Cere_Crus2, cerebellum_crus2; CN, caudate nucleus; L (R), left (right) hemisphere; PD, Parkinson’s disease; PPS, Parkinsonism-Plus syndrome; HC, healthy controls. *p < 0.05; **p < 0.01; ***p < 0.001

CBFs in seven central regions were significantly different among the PD, PPS, and HC groups (GRF correction, voxel-level threshold *p* < 0.01, cluster level threshold *p* < 0.05). The specific locations of the brain regions are listed in Table 2 and Fig. 1**.** The further post-hoc analysis showed that PD group had a significantly decreased CBF in the left frontal medial orbital gyrus (FG_Med_Orb), the left triangle inferior frontal gyrus (IFG_Tri), the left middle frontal gyrus (MFG), the right cerebelum_crus2 and the left caudate nucleus (CN), compared with the HC group (*p* < 0.05). Besides the above regions, the left supplementary motor area (SMA), the right thalamus showed decreased CBF in the PPS group compared with the HC group (*p* < 0.05). PPS group had lower CBF value in the left IFG_Tri, the left MFG, the left CN, the left SMA, and the right thalamus compared with the PD group (*p* < 0.05).

**Table 2:** CBF in different brain regions among the PD, PPS, and HC groups

Peak location (AAL-90)	MNI coordinate			No. of voxels	Peak *F*-value	HC	PD	PPS	PD-HC	PPS-HC	PPS-PD
	x	y	z			Mean/SD	Mean/SD	Mean/SD	*p*-value	*p*-value	*p*-value
SMA.L	− 2	24	60	481	3.73	43.54/11.98	42.59/10.87	29.04/10.15	0.794	**< 0.001**	**0.001**
THA.R	1	−16	5	313	3.74	58.83/12.32	54.01/11.62	42.38/12.21	0.229	**< 0.001**	**0.006**
FG_Med_Orb.L	0	64	−4	646	3.82	57.03/11.38	47.94/12.05	40.56/9.34	**0.016**	**< 0.001**	0.053
IFG_Tri.L	−52	20	2	580	3.88	46.62/4.65	41.28/9.29	35.15/7.07	**0.034**	**< 0.001**	**0.017**
MFG.L	−40	26	40	377	3.93	48.80/7.25	41.58/11.46	33.62/9.67	**0.029**	**< 0.001**	**0.018**
Cere_Crus2.R	50	−66	−50	622	4.25	31.52/10.35	19.45/13.53	14.58/6.15	**0.001**	**< 0.001**	0.182
CN. L	−10	4	12	457	4.56	33.61/5.34	29.48/4.19	24.93/6.11	**0.020**	**< 0.001**	**0.012**

Abbreviations: CBF, cerebral blood flow; AAL, automated anatomical labeling; HC, healthy controls; PD, Parkinson’s disease; PPS, Parkinsonism-Plus syndrome; SMA, supplementary motor area; THA, thalamus; FG_Med_Orb, frontal medial orbital gyrus; IFG_Tri, triangle inferior frontal gyrus; MFG, middle frontal gyrus; Cere_Crus2, cerebellum_crus2; CN, caudate nucleus; L (R), left (right) hemisphere; SD, standard deviation

### ROC curve analysis

**Fig. 2:**
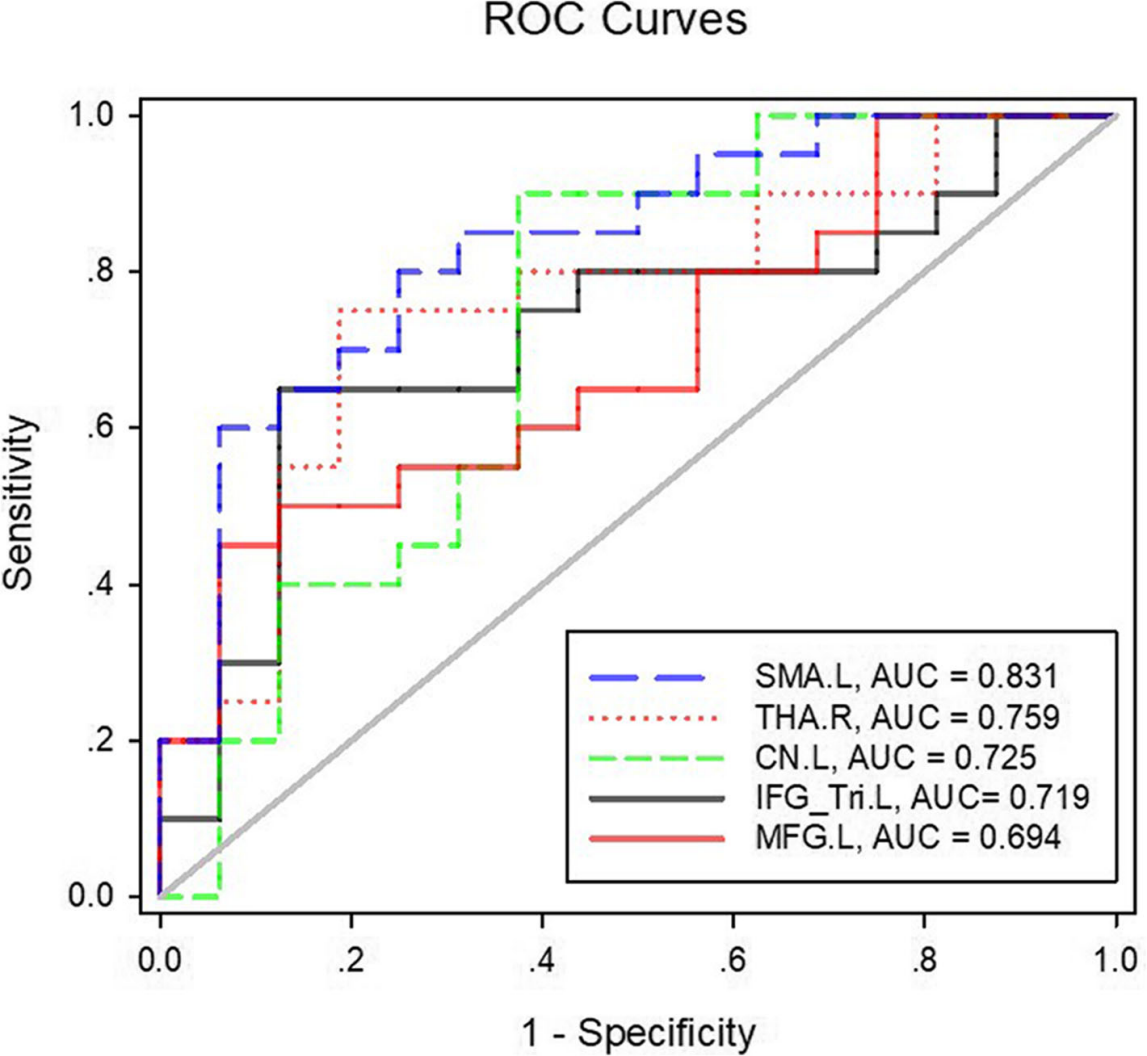
ROC curves of CBF in the regions with a significant between-group difference for distinguishing PD patients from PPS patients. The diagonal line (grey line) represents the area under the curve of 0.50. Abbreviations: ROC, receiver operating characteristic; SMA, supplementary motor area; THA, thalamus; CN, caudate nucleus; IFG_Tri, triangle inferior frontal gyrus; MFG, middle frontal gyrus; L (R), left (right) hemisphere; PD, Parkinson’s disease; PPS, Parkinsonism-Plus syndrome

ROC curve analysis of CBF values in the left MFG, the left IFG_Tri, the left CN, the left SMA, and the right thalamus is shown in Table 3 and Fig. 2. CBF of the left SMA achieved a highest area under the curve (AUC) of 0.831 (*p* = 0.001), followed by CBF of the right thalamus (AUC: 0.759, *p* = 0.008), CBF of the left CN (AUC: 0.725, *p* = 0.022), CBF of the left IFG_Tri (AUC: 0.719, *p* = 0.026), and CBF of the left MFG (AUC: 0.694, *p* = 0.048).

**Table 3:** ROC analysis of CBF for differentiation between the PD and PPS groups

Brain region	AUC	Cutoff point	Sensitivity (%)	Specificity (%)	95% CI		*p*-value
					Upper bound	Lower bound	
SMA.L	0.831	33.723	0.800	0.750	0.696	0.967	0.001
THA.R	0.759	50.195	0.750	0.812	0.597	0.922	0.008
CN. L	0.725	24.544	0.900	0.625	0.547	0.903	0.022
IFG_Tri.L	0.719	39.483	0.650	0.875	0.545	0.892	0.026
MFG.L	0.694	44.581	0.550	0.937	0.521	0.866	0.048

Abbreviations: ROC, receiver operating characteristic; PD, Parkinson’s disease; PPS, Parkinsonism-Plus syndrome; AUC, area under the curve; CI, confidence interval; SMA, supplementary motor area; THA, thalamus; CN, caudate nucleus; IFG_Tri, triangle inferior frontal gyrus; MFG, middle frontal gyrus; L (R), left (right) hemisphere

### Correlations between CBF values and clinical scores

The results of correlation analysis are shown in Table 4. The CBFs of left MFG, the left CN, the right thalamus, and the right cerebelum_crus2 were positively correlated with MoCA scores (*r =* 0.584, 0.548, 0.723, 0.499, respectively; all *p* < 0.05) and the CBFs of the right thalamus as well as the left CN were positively correlated with MMSE scores in PD patients (*r =* 0.745, 0.464, respectively; all *p* < 0.05) in PD patients. The CBF of left IFG_Tri was positively correlated with the MMSE score (*r =* 0.783; *p* = 0.004) and the CBF of the right cerebellum_crus2 was positively correlated with HAMA and HAMD scores (*r =* 0.817, 0.664, respectively; all *p* < 0.05) in PPS patients.

**Table 4:** Correlation between CBF values and clinical scores in PD and PPS groups

	HAMA		HAMD		MMSE		MoCA	
	*r*	*p*	*r*	*p*	*r*	*p*	*r*	*p*
PD group								
SMA.L	0.035	0.883	0.101	0.672	0.370	0.108	0.501	0.024
THA.R	−0.155	0.515	−0.157	0.509	0.745	**< 0.001**	0.723	**< 0.001**
FG_Med_Orb.L	−0.158	0.505	− 0.163	0.492	0.257	0.273	0.384	0.095
IFG_Tri.L	−0.271	0.248	−0.171	0.472	0.296	0.205	0.423	0.063
MFG.L	−0.042	0.862	0.020	0.932	0.425	0.062	0.584	**0.007**
Cere_Crus2.R	−0.087	0.716	−0.205	0.387	0.220	0.351	0.499	**0.025**
CN.L	−0.024	0.920	−0.032	0.894	0.464	**0.039**	0.548	**0.012**
PPS group								
SMA.L	−0.095	0.781	−0.019	0.955	0.422	0.196	0.201	0.553
THA.R	0.053	0.878	0.299	0.372	0.267	0.427	0.179	0.599
FG_Med_Orb.L	−0.355	0.284	−0.090	0.793	0.592	0.055	0.307	0.359
IFG_Tri.L	−0.261	0.438	−0.088	0.798	0.783	**0.004**	0.539	0.087
MFG.L	−0.599	0.074	−0.192	0.571	0.399	0.224	0.217	0.522
Cere_Crus2.R	0.817	**0.002**	0.664	**0.026**	−0.043	0.901	0.034	0.921
CN.L	−0.218	0.520	−0.061	0.859	0.376	0.255	0.105	0.759

Abbreviations: CBF, cerebral blood flow; PD, Parkinson’s disease; PPS, Parkinsonism-Plus syndrome; SMA, supplementary motor area; THA, thalamus; FG_Med_Orb, frontal medial orbital gyrus; IFG_Tri, frontal inferior, triangle gyrus; MFG, frontal middle gyrus; Cere_Crus2, cerebellum_crus2; CN, caudate nucleus; L (R), left (right) hemisphere; HAMA, Hamilton Anxiety Scale; HAMD, Hamilton Depression Scale; MMSE, Mini-Mental State Examination; MoCA, Montreal Cognitive Assessment

## Discussion

Our study results showed that both PD and PPS patients had reduced CBF in several brain regions compared with healthy controls. PPS patients had lower CBF in the left MFG, the left IFG_Tri, the left CN, the left SMA, and the right thalamus than PD patients. CBF value in these five brain regions had a desirable performance for discrimination between PD and PPS.

Previously, arterial spin labeling imaging combined with diffusion tensor imaging has been shown as useful markers for early Parkinson’s disease [29]. In our study, PD patients showed reduced CBF in five brain regions, i.e., the right cerebelum_crus2, the left MFG, the left IFG_Tri, the left FG_Med_Orb, and the left CN, as detected by 3D-pCASL. These results were consistent with previous studies using PET/SPECT and ASL [23, 30–33], where hypoperfusion was found in widespread cortical regions [20, 23], particularly in frontal regions [23, 30–32], as well as CN [23], cerebellar regions in PD patients [33]. These areas are often associated with motor function, and cognitive impairments or depression in PD [34, 35]. In addition, hypoperfusion in the left CN and the right cerebelum_crus2 was found in PD patients in our study. Previous studies using ASL technique also found hypoperfusion in the right and left CN in patients with PD [23] and the CBF laterality pattern in the CN was a biomarker for PD diagnosis [36]. The hypoperfusion of CN maybe relate to the dopamine loss, which is associated with the cognitive decline [37] and depressive symptoms [38] in PD. Our results also showed a positive correlation between the CBFs of the left MFG and the left CN and non-motor neuropsychological scores including MMSE and MoCA scores in PD patients. This indicated that the CBF reduction might be associated with cognition decline in the PD. The cerebellum is an important component in motor control. To date, contradictory results have been reported for the cerebellum CBF change in the PD, the exact role of the cerebellum in PD remains to be further understood [33].

It is challenging to differentiate the early-middle stage of PPS from PD based on clinical symptoms. Previously, SPECT and PET-CT have been used to discriminate PD from parkinsonian disorders, such as Parkinson variant of MSA and PSP [39, 40] based on a distinct hypoperfusion pattern in the frontal cortex, thalamus, and cerebellum [39]. In our study, PPS patients showed reduced CBF in five brain regions similar to those of PD and in two additional brain regions, i.e., the left SMA and the right thalamus, as detected by 3D-pCASL. Comparatively, PPS patients had altered CBF in more widespread brain regions. The additional involvement of the left SMA and the right thalamus suggests that PPS patients might have more severe impairment of motor function than PD patients. Further correlation analysis showed that the CBF of left IFG_Tri was positively correlated with the MMSE score and the CBF of the right cerebellum_crus2 was positively correlated with HAMA and HAMD scores in PPS patients. The association between the hypoperfusion in the cerebellum, thalamus, SMA and the damage in the extrapyramidal system has been described in PPS patients [41]. Thus, more widespread and severe impairment of CBF in PPS might reflect more severity of PPS or later stage of disease.

Our study showed that PPS patients had lower CBF values in the left IFG_Tri, the left MFG, the left CN, the left SMA, and the right thalamus compared with PD patients. This different pattern of reduced brain perfusion between PPS and PD is consistent with the previous study, where the perfusion distribution patterns were found to be different among MSA-P, PSP, and PD [42]. In our study, further ROC analysis showed that CBF value in these five brain regions had a desirable AUC for discrimination between PD and PPS, with CBF of the left SMA achieving the highest AUC of 0.831. Previously, a multitude of imaging parameters have been used to differentiate between PD and PPS [9, 43–45]. Tir et al. found that PSP had a lower fractional anisotropy value derived from diffusion tensor imaging in the SMA [46]. Calloni et al. reported that the middle cerebellar peduncle width and putaminal hypointensity on susceptibility-weighted imaging (SWI) can be used in combination to distinguish atypical parkinsonisms from idiopathic PD, with an AUC = 0.98 [43]. In addition, swallow tail (AUC = 0.85) and putaminal hypointensity (AUC = 0.68) also were reported to be able to distinguish MSA from PD [9]. Comparatively, the participants in these studies were older and had longer disease duration. In our study, PPS patients in the earlier stage of the disease were included. Our results showed the CBF of the SMA had the greatest AUC (0.831) and might be used as a surrogate marker for the differential diagnosis between the two diseases in an earlier stage.

There are some limitations to this study. First, we did not investigate CBF in early PD patients. The CBF as detected by ASL has been reported in early PD patients and perfusion reduction did not differ among different stages of PD [29]. Second, the sample size is small. Third, the numbers of clinical scales collected are insufficient. Specifically, Unified Parkinson’s Disease Rating Scale (UPDRS) as a rating tool to assess motor symptoms of PD patients was not applied in our study to evaluate the motor symptoms in PPS patients. Thus, motor clinical scores were not available for all patients. Third, a single PLD time was used in our study. Although the PLD of 1525 ms was commonly used in previous ASL studies [20, 47]. However, a fixed PLD could bring the risk of inaccurate estimation of the CBF when the PLD used is shorter or longer than the arterial transit time. Future studies with multiple PLDs are needed to validate the discriminative CBF in these two diseases. Finally, CBF was measured in each patient under medication in “on” state. The effect of medication state on CBF requires further investigation.

## Conclusion

In conclusion, our study demonstrated that PD and PPS patients showed hypoperfusion in several brain regions and PPS showed severer and more widespread impairment in CBF. PD and PPS patients have a certain discriminative pattern of reduced CBFs, in particular, reduced CBF in left SMA, which can be used as a surrogate marker for differential diagnosis.

## Data Availability

The datasets generated and/or analysed during the current study are available from the corresponding author on reasonable request.

## Abbreviations

PD: Parkinson’s disease
PPS: Parkinsonism-Plus syndrome;
MSA: Multiple system atrophy
PSP: Progressive supranuclear palsy
CBD: Corticobasal degeneration
MRI: Magnetic resonance imaging
PET: Positron emission tomography
SPECT: Single-photon emission computed tomography
ASL: Arterial spin labeling
3D-pCASL: Three-dimensional pseudo-continuous arterial spin labeling
CBF: Cerebral blood flow
HCs: Healthy controls
HAMA: Hamilton Anxiety Scale
HAMD: Hamilton Depression Scale
MMSE: Mini-Mental State Examination
MoCA: Montreal Cognitive Assessment
TR: Repetition time
TE: Echo time
TI: Inversion time
FA: Flip angel
FOV: Field of view
VBA: Voxel-based analysis
MNI: Montreal Neurological Institute
SNR: Signal-to-noise ratio
FWHM: Full-width at half-maximum
ANOVA: One-way analysis of variance
GRF: Gaussian Random Field
AAL: Anatomical Automatic Labeling
LSD: Least significant difference test
ROC: Receiver operating characteristic
FG_Med_Orb: Frontal medial orbital gyrus
IFG_Tri: Triangle inferior frontal gyrus
MFG: Middle frontal gyrus
CN: Caudate nucleus
SMA: Supplementary motor area
AUC: Area under the curve
SWI: Susceptibility-weighted imaging
PLD: Post-labeling delay
